# Potential role of PCTAIRE-2, PCTAIRE-3 and P-Histone H4 in amyloid precursor protein-dependent Alzheimer pathology

**DOI:** 10.18632/oncotarget.7380

**Published:** 2016-02-14

**Authors:** Dale Chaput, Lisa Kirouac, Stanley M. Stevens, Jaya Padmanabhan

**Affiliations:** ^1^ Department of Cell Biology, Microbiology, and Molecular Biology, University of South Florida, Tampa, FL, USA; ^2^ Department of Molecular Medicine, USF Health Byrd Alzheimer's Institute, University of South Florida, Tampa, FL, USA

**Keywords:** Alzheimer's disease, amyloid precursor protein, phosphoproteomics, A-beta, neurodegeneration, Gerotarget

## Abstract

Amyloid Precursor Protein (APP) is regulated in a mitosis-specific manner and plays a role in proliferative signaling in cells. Though APP-derived Aβ generation has a well-established role in neurodegeneration, the mechanistic role of APP in this process is not fully understood. Here, we performed an unbiased, comprehensive analysis of the phosphoproteome signature in APP-null neuroblastoma cells (B103) compared to those expressing APP-695 isoform (B103-695) to determine if APP expression affects protein phosphorylation. Stable isotope labeling by amino acids in cell culture (SILAC) followed by mass spectrometry-based phosphoproteomic analysis with PolyMAC identified a total of 2,478 phosphopeptides in the B103 and B103-695 cell culture model system. We observed that phosphorylation of PCTAIRE-2 (CDK17), PCTAIRE-3 (CDK18), and Histone H4 are significantly elevated in B103-695 cells; western blot analysis confirmed overexpression of PCTAIREs and increased phosphorylation of Histone H4. More importantly, analysis of primary neurons treated with Aβ, as well as brain samples from MCI (mild cognitive impaired) and AD patients recapitulated these results, showing increased levels of PCTAIREs and P-Histone H4. These novel findings identify a hitherto uncharacterized mechanism by which APP and/or Aβ may promote AD neurodegeneration, and raises the possibility that their inhibition may protect against pathology development in AD.

## INTRODUCTION

Alzheimer's disease (AD) is the most prevalent form of dementia and causes deficits in memory and executive function. A central lesion in AD is the extracellular amyloid plaque, composed of aggregated Aβ peptide that accumulates in the brain before onset of disease symptoms [1]. Aβ is generated upon sequential cleavage of amyloid precursor protein (APP) by β-secretase and γ-secretase [2, 3]. When APP is cleaved by α-secretase within the Aβ domain, it liberates sAPPα and precludes the generation of Aβ [3]. Since APP expression leads to generation of not only full length APP but also APP fragments with various cellular functions, it is difficult to determine how the APP holoprotein and its metabolites differentially contribute to AD.

While genetic factors contributing to familial AD (FAD) are well described, little is known about the molecular processes leading to disease state in sporadic AD (SAD). In both subsets of AD, Aβ accumulation precedes formation of neurofibrillary tangles (NFTs), highlighting the role of Aβ in disease pathogenesis [4]. In early stages of AD, Aβ fibrils and oligomers induce microgliosis [5, 6] and this immune response can induce abnormal cell cycle events in compromised neurons of the AD brain [7-11]. Our earlier studies demonstrated that mouse models of AD, expressing APP alone or together with Presenilin 1 (PS1), show aberrant expression of cell cycle regulatory proteins with concomitant increase in phosphorylation of APP at Thr668 and association of P-APP with the centrosomes [12]. Phosphorylation of APP at this site is associated with altered APP trafficking and enhanced proteolysis by β-secretase, [13-16]. Since Aβ is known to induce neuronal cell cycle entry [7, 17], this will further enhance APP phosphorylation and β-secretase-mediated proteolysis to generate more Aβ, thereby feeding back to the vicious cycle of neurodegeneration.

Our published study, showing that APP promotes expression of proliferation-associated proteins, supports the notion that APP has a cell cycle regulatory function [18]. This study was carried out with B103 rat neuroblastoma cells that are null for or expressing the APP-695 (B103-695) isoform [19], using Stable Isotope Labeling by Amino Acids in Cell Culture (SILAC)-based mass spectrometry. These findings indicate that APP expression induces signaling cascades that may be involved in cell cycle-mediated neuronal degeneration observed in AD. Since cell cycle deregulation and protein phosphorylation are fundamental to AD neurodegeneration, in the current study we analyzed global changes in protein phosphorylation upon APP expression using mass spectrometry-based phosphoproteomics. Mass spectrometry was used for identification of phosphopeptides from complex mixtures, including site localization and relative quantification. Phosphoproteomics involves enrichment of phosphopeptides to increase identification and sequence information, which is useful for bioinformatic analysis of affected pathways. Phosphoproteomics can identify potential changes in kinase activity by analysis of overrepresented phosphorylated consensus motifs [20]. Several phosphopeptide enrichment techniques are described, including antibody immunoaffinity enrichment, immobilized metal-affinity chromatography (IMAC) using Fe^3+^ metal ions [21-23], and metal oxide affinity chromatography (MOAC) using TiO_2_ [24, 25]. Currently, there are few phosphoproteomic studies related to AD. Here, we used a titanium-based nanopolymer phosphopeptide enrichment in combination with strong cation exchange (SCX). Phosphoproteomic analysis identified over 2000 phosphopeptides in SILAC-labeled B103 and B103-695 cells. Targeted validation of selected signaling markers in PS/APP mice, Aβ-treated primary neurons and human AD and MCI patient brain tissue provided insights into novel biomarkers associated with AD pathology.

## RESULTS

### B103 and B103-695 phosphoproteome comparison reveals differential phosphorylation of proteins in APP expressing cells

A total of 2478 phosphopeptides were identified among 3 biological replicates in B103 and B103-695 cells; 1082 were quantified in a minimum of 2 biological replicates with a minimum ratio count of 2. Of the 1082 phosphorylation sites confidently identified and quantified, 712 of them corresponded to proteins previously quantified by SILAC in our global scale proteomic analysis of B103 and B103-695 cells [18]. When possible, phosphosite ratios were normalized against corresponding protein ratios previously determined in the B103 and B103-695 proteomic analysis. Perseus was used to identify significant changes in phosphopeptide expression across biological replicates using Significance A, a statistical outlier test, with a *p*-value threshold of 0.05. Significant changes were identified in 92 phosphosites corresponding to 71 different proteins when using non-normalized ratios, and 50 phosphosites corresponding to 46 proteins when using normalized ratios. Selected differentially phosphorylated sites, both normalized and non-normalized, are listed in Table 2. Bioinformatic analysis of statistically significant phosphosites was performed using Ingenuity Pathway Analysis (IPA) which identified several proteins associated with neurological disease and psychological disorders as well as molecular and cellular functions including cell morphology, cellular assembly and organization, function and maintenance, and growth and proliferation.

Our phosphoproteomic analysis identified a number of differentially phosphorylated proteins that have been previously associated with AD, including A-kinase anchor protein 12 (AKAP12), heat shock protein beta 1 (HspB1) and myristoylated alanine-rich C-kinase substrate (MARCKS). AKAP 12 exhibited significantly increased phosphorylation at Ser507 (2.46-fold) in B103-695 cells after normalization against total protein expression. AKAP12 is a scaffolding protein that serves as a negative regulator of G1 to S cell cycle progression [26]. Increased phosphorylation of AKAP12 at Ser290 was also observed in an early phosphoproteomic study of human AD brain [27], however, the functional relevance of this residue is not known. B103-695 cells also showed increased phosphorylation of HspB1, a molecular chaperone involved in regulating cell proliferation and cytoskeletal reorganization at Ser15 (3.16-fold) and Ser86 (4.49-fold) [28-30]. A recent quantitative phosphoproteomic study of frontal cortex from human AD brains also identified increased phosphorylation of HspB1 at these sites [31]. MARCKS showed significant decrease in phosphorylation at several sites in B103-695 cells, however these phosphopeptide ratios were unable to be normalized against total protein levels. A previous study showed that human AD cortical neurons exhibited an overall decrease in MARCKS phosphorylation, however they also reported an increase in phosphorylation of this protein in microglia from AD brains [32]. Taken together, we have identified a number of differentially phosphorylated proteins that have previously been reported to show altered phosphorylation in AD, which provides confidence in our cell model for APP-related studies as well as the quality of our phosphoproteomic dataset.

### Consensus motif analysis of identified phosphorylation sites

**Figure 1:**
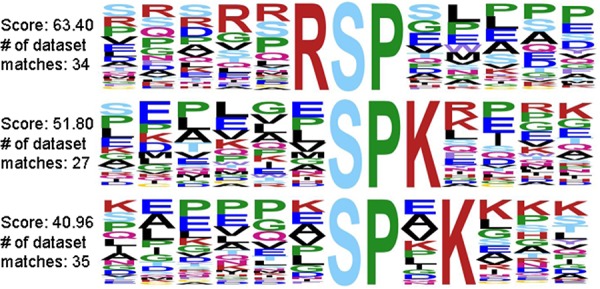
Growth-associated Histone H1 kinase substrate motif represented in APP-695 expressing B103-695 cells but not in APP-null B103 cells

Mass spectrometry provides site localization of phosphorylated peptides as well as surrounding sequence information, which enables consensus motif analysis. Most kinases phosphorylate residues within a specific consensus motif; determining overrepresented consensus motifs can be an indication of changes in kinase activity. Consensus motif analysis identified several phosphopeptides that were phosphorylated within the growth associated Histone H1 kinase substrate motif in B103-695 cells but not in B103 cells, suggesting increased activity of this kinase in APP-695 expressing cells (Figure 1). Growth-associated Histone H1 kinase has been shown to be involved in regulating mitotic entry [33], suggesting altered cell cycle regulation in B103-695 cells.

### APP expressing cells show increased phosphorylation of Histone H4 at Ser47

**Figure 2:**
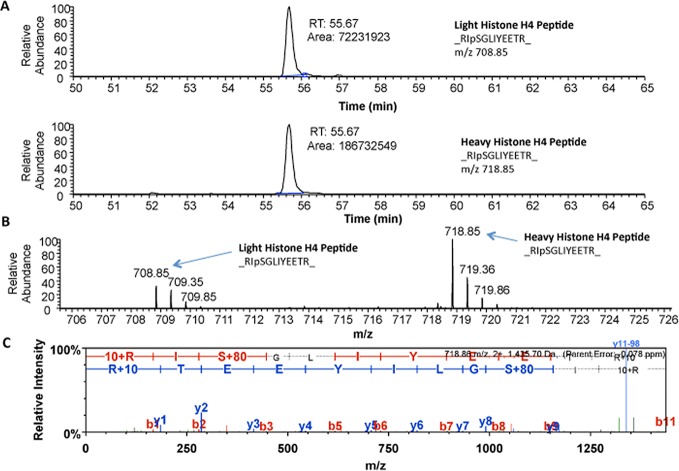
Mass spectrometry identified increased phosphorylation of Histone H4 at Ser47 in B103-695 cells **A.** Extracted Ion Chromatogram for “Light” (top) and “Heavy” (bottom) Histone H4 peptide RpSGLIYEETR. **B.** Base peak chromatogram showing isotope clusters for both “Light” and “Heavy” peptides with monoisotopic masses labelled. **C.** Annotated MS/MS spectra of Histone H4 peptide showing phosphorylation detected at Ser47.

**Figure 3:**
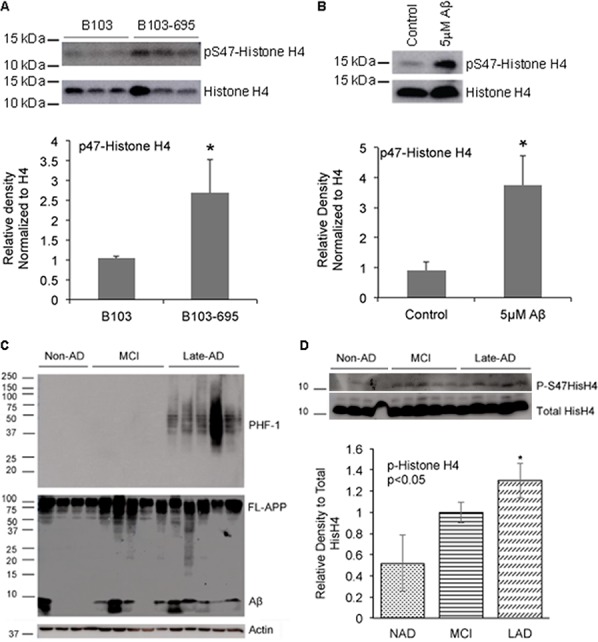
APP expressing cells, Aβ-treated neurons and AD samples show increased phosphorylation of Histone H4 at Serine 47 **A.** B103-695 cells show increased phosphorylation of Ser47 Histone H4: Nuclear fractions isolated from B103 and B103-695 cells were analyzed using P-Histone H4 Ser47 (top panel) antibodies. Blot was reprobed with total Histone H4 antibody for normalization (bottom panel). The bar graph shows ratio of P-Histone H4 to total Histone H4. **B.** Neurons treated with Aβ show increased phosphorylation of Histone H4 at Ser 47: Primary cortical neurons were treated with 5μM Aβ for 24hr and samples were analyzed by western blot using Ser 47 P-Histone H4 antibody (top panel). Blots were reprobed with total Histone H4 antibody for normalization (bottom panel). The bar graph shows ratio of P-Histone H4 to total histone H4. **C.** Human MCI and AD samples show increased levels of P-Histone H4 compared to non-AD: Equal amounts of proteins from human brain samples (shown in Table 2) analyzed using PHF-1 (top panel) and 6E10 (middle panel) antibodies show increased levels of P-tau (top panel) and Aβ (middle panel) in LAD samples compared to MCI and non-AD. Actin antibody was used for normalization (bottom panel). **D.** P-Histone H4 Ser47 is significantly increased in LAD brain samples: Blots from the human samples were reprobed with P-Histone H4 (top panel) and total Histone H4 (bottom panel) antibodies, which showed a tendency towards increase in Ser47 P-Histone H4 in MCI and significant increase in LAD; bar graph shows ratio of P-Histone H4 to total Histone H4. *p value < 0.05.

The normalized ratio of phosphoSer47-Histone H4 showed a statistically significant 1.89-fold increase in B103-695 cells compared to B103 cells and was selected for further validation. Histone H4 is involved in chromatin structure and function and modification of Histone H4 influences both dynamic and long-term gene expression. Histone H4 is phosphorylated at Ser47 by p21-protein-activated kinase 2 (Pak2) [34]. Pak2 is activated upon phosphorylation at Ser141 [35]. We observed a slight increase in phosphorylation of Pak2 at Ser141 (1.15-fold increase after normalization to total Pak2) in B103-695 cells by phosphoproteomics (data not shown). The extracted ion chromatogram (XIC) for the SILAC heavy and light labeled Ser47 phosphorylated Histone H4 peptide identified by LC-MS/MS analysis, as well as their base peak chromatograms, are shown in Figure 2A and 2B, respectively. The area under the curve for each XIC is representative of peptide abundance, which is significantly greater in the heavy labeled peptides from B103-695 cells. The annotated MS/MS spectra showing the amino acid sequence determined by LC-MS/MS of the Ser47 phosphorylated Histone H4 peptide is shown in Figure 2C. Increased phosphorylation of Ser47-Histone H4 in B103-695 cells was validated by western blot analysis of nuclear fractions from B103 and B103-695 samples (Figure 3A).

Since we observed significant changes in Histone H4 phosphorylation in APP expressing cells, we evaluated if similar changes occur in Aβ treated neurons and human AD brain samples. Primary cortical neurons were treated with 5μM oligomeric Aβ42 for 24 hours, and analyzed using the phospho-specific Histone H4 antibody, which showed a significant increase in phosphorylation of Histone H4 at Ser47 while total Histone H4 levels were unaltered (Figure 3B). Next we tested if similar changes in Histone H4 phosphorylation occur in the MCI or AD brain samples. Western blot analysis with Aβ-directed 6E10 and PHF-1 (Ser396/Ser404) P-tau antibodies were performed to validate that the MCI and LAD brain samples indeed show increased levels of Aβ and/or hyperphosphorylation of tau compared to the NAD samples (Figure 3C; specifications of the samples are provided in Table 1). Reprobe of the blots with the Ser47-specific P-Histone H4 antibodies showed an increase in P-Histone H4 levels in MCI, with significant increase in LAD, compared to the NAD samples (Figure 3D). Total levels of H4 appeared to be unaltered between the various brain samples. These data imply that phosphorylation of Histone H4 at Ser47 is a disease-specific modification and this might have implications in advancement of pathology development in AD.

**Table 1:** Specifications on human brain tissue samples

Case No.	Age	Sex	Braak Stage	PMI	MMSE	Diagnosis
34	91	F	3	3.33	30	NAD
29	83	F	4	5.25	30	NAD
41	91	F	4	4.82	29	NAD
40	91	F	2	3.8	29	NAD
24	86	F	3	2.92	22	MCI
17	86	F	3	6.17	30	MCI
9	87	M	5	6.17	24	MCI
35	94	M	1	3.87	27	MCI
45	95	F	5	5.30	24	MCI
12	82	F	6	5.92	17	LAD
39	90	M	6	4.17	14	LAD
37	88	F	5	4.50	10	LAD
10	82	F	6	4.58	−5	LAD
40	96	F	6	4.50	20	LAD

Details of the brain samples used in the study are provided in this table. PMI indicates post mortem interval. MMSE indicates clinical assessment using The Mini Mental State Examination. NAD: Non-Alzheimer's Disease (normal samples); MCI: Mild Cognitive Impairment; LAD: Late-stage Alzheimer's disease.

### Increased expression of PCTAIRE-2 and PCTAIRE-3 in APP-expressing cells

**Figure 4:**
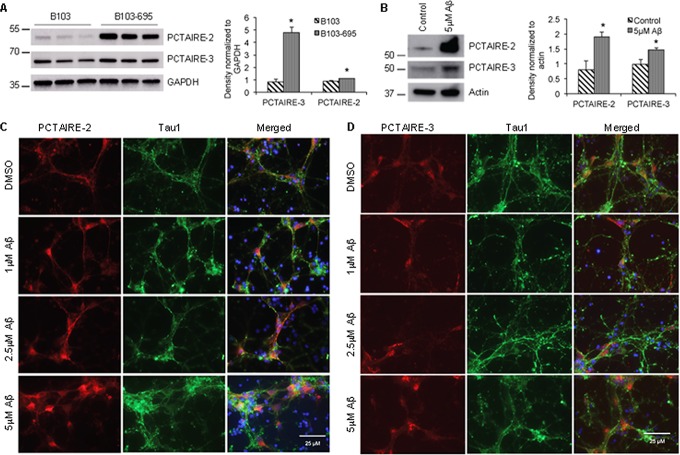
APP expression and Aβ treatment increase the levels of PCTAIRE-2 and PCTAIRE-3 in cells Western blot analysis shows that PCTAIRE-2 and PCTAIRE-3 are significantly increased in **A.** B103-695 cells compared to B103 cells and **B.** primary neurons treated with Aβ compared to vehicle treated controls. The bar graphs to the right of the blots show data normalized to GAPDH levels in B103 and B103-695 cells and actin in primary neurons. *p value < 0.05. **C.** and **D.** Primary cortical neurons were treated with or without 1, 2.5 and 5 μM Aβ for 24 hr and co-immunostaining analysis was performed using (C) PCTAIRE-2 or (D) PCTAIRE-3 and Tau1 antibodies. Hoechst was used to visualize the nuclei. Neurons treated with Aβ show increased nuclear and perinuclear staining.

Phosphopreoteome analysis showed that PCTAIRE-2 (CDK17) and PCTAIRE-3 (CDK18), members of the cyclin-dependent kinase (Cdk) family, are differentially phosphorylated in B103-695 cells compared with B103 cells (Table 2). PCTAIRE-2 and PCTAIRE-3 were not identified in our initial proteomic analysis of B103 and B103-695 cells [18], but their non-normalized phosphosite ratios showed significant increases in phosphorylation. PCTAIRE-2 showed increased phosphorylation at Ser146 (1.86-fold) and Ser180 (2.27-fold). PCTAIRE-3 showed increased phosphorylation at Ser66 (3.5-fold) and Ser109 (3.85-fold). Western blot analysis revealed that expression of both PCTAIRE-2 and PCTAIRE-3 were significantly increased in B103-695 cells compared to B103 cells (Figure 4A).

**Table 2:** Selected proteins that showed significant change in phosphorylation in B103-695 compared to B103

Protein Name	Gene Name	Protein	Amino Acid	Pos.	Median Ratio	Variance	Median Protein Ratio	Normalized Median
Heat shock protein beta-1	Hspb1	P42930	S	86	2.90	0.35	0.64	4.50
Heat shock protein beta-1	Hspb1	P42930	S	115	2.04	0.08	0.64	3.16
A-kinase anchor protein 12	Akap12	Q5QD51	S	507	1.79	0.36	0.73	2.46
Cyclin-dependent kinase 17	Cdk17	O35381	S	146	1.86	0.21	N/A	N/A
Cyclin-dependent kinase 17	Cdk17	O35381	S	180	2.27	0.92	N/A	N/A
Cyclin-dependent kinase 18	Cdk18	O35382	S	109	3.85	0.15	N/A	N/A
Cyclin-dependent kinase 18	Cdk18	O35382	S	66	3.5	0.099	N/A	N/A
Histone H4	Hist1h4b	P62804	S	47	2.24	0.018	1.18	1.90
Myristoylated alanine-rich C-kinase substrate	Marcks	P30009	T	143	0.39	0.002	N/A	N/A
Myristoylated alanine-rich C-kinase substrate	Marcks	P30009	S	27	0.25	6.47E-4	N/A	N/A
Myristoylated alanine-rich C-kinase substrate	Marcks	P30009	S	138	0.26	2.17E-5	N/A	N/A
Cell division cycle protein 20	Cdc20	Q62623	T	106	2.15	0.004	0.96	2.25

Selected phosphoproteins of interest that showed significant change in phosphorylation in B103-695 cells compared to the APP-null B103 cells and the amino acid position (Pos.) are provided in this table. Normalized and non-normalized phosphosite ratios were analyzed using Significance A with a Benjamini-Hochberg q-value cutoff of 0.05, protein ratios with q-values ≤ 0.05 in two out of three biological replicates were considered significant.

### Aβ treated neurons show altered cellular distribution and enhanced expression of PCTAIRE-2 and PCTAIRE-3

To determine if Aβ affects expression or cellular distribution of the PCTAIRE proteins we next analyzed primary neurons treated with or without Aβ. Cortical neurons were treated with oligomeric Aβ42 for 24 hours and analyzed by western blot and immunostaining. Western blot analysis revealed a significant increase in levels of both PCTAIRE-2 and PCTAIRE-3 following treatment with 5μM Aβ for 24 hours (Figure 4B). Immunostaining of these neurons treated with 1μM, 2.5μM, and 5μM oligomeric Aβ42 showed dose-dependent alteration of PCTAIRE-2 and PCATIRE-3 expression and cellular distribution. Control neurons treated with DMSO exhibited basal, cytoplasmic staining of PCTAIRE-2 whereas those treated with even the lowest concentration of Aβ showed enhanced PCTAIRE-2 staining that accumulates both in the nuclear and perinuclear areas (Figure 4C). PCTAIRE-3 staining in the neurons showed reduced levels compared to that of PCTAIRE-2, agreeing with our western blot analysis. In control DMSO treated neurons, PCTAIRE-3 exhibited basal, punctate nuclear staining (Figure 4D, top row). Upon Aβ treatment, expression of PCTAIRE-3 was increased, as indicated by enhanced staining, which appeared to localize to not only nuclear regions, but also to the cell body in a fibrillar pattern (Figure 4D).

### Aβ-mediated induction in PCTAIRE-2 and PCTAIRE-3 is dependent on APP expression

**Figure 5:**
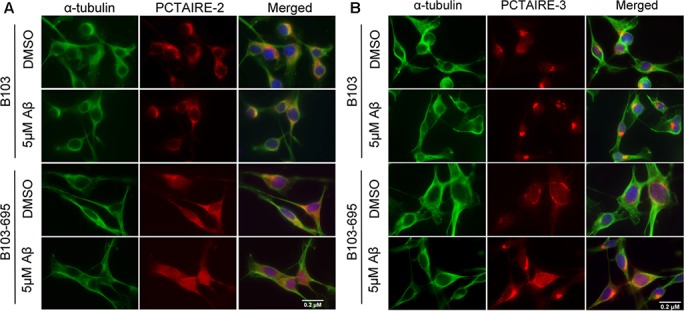
Aβ affects PCTAIRE-2 and PCTAIRE-3 cellular distribution in an APP-dependent manner B103 and B103-695 cells were treated with 5μM Aβ for 24hr and immunostaining analysis was performed using **A.** PCTAIRE-2 or **B.** PCTAIRE-3 and α-tubulin antibodies. Hoechst was used to visualize the nuclei. We observed an increase in nuclear staining in B103-695 cells following Aβ treatment, while APP-null B103 cells did not show any significant change in staining.

Aβ-mediated cell toxicity has been shown to depend on APP expression on the cellular membrane [36]. To examine both the independent and concerted roles of Aβ and APP in inducing expression of PCTAIRE-2 and 3, we treated B103 and B103-695 cells with 5μM oligomeric Aβ42 for 24 hours and analyzed for changes in expression and localization of PCTAIREs. DMSO (vehicle) treated B103 cells appeared to have focused, perinuclear staining with PCTAIRE-2, and Aβ treatment did not appear to affect the staining in the perinuclear area (Figure 5A, top 2 rows). B103-695 cells treated with vehicle showed staining for PCTAIRE-2 spread throughout the cell and, upon Aβ treatment, appeared to show increased staining in the nucleus (Figure 5A, bottom 2 rows). Similar to PCTAIRE-2, PCTAIRE-3 also showed slight alterations in staining in B103 cells exposed to Aβ, with the vehicle-treated control cells showing condensed perinuclear staining, which became more compact and intense after Aβ treatment (Figure 5B, top 2 rows). In B103-695 cells, vehicle treated cells showed perinuclear PCTAIRE-3 staining with light punctate staining in the nucleus (Figure 5B, bottom 2 rows). This staining became significantly enhanced upon Aβ treatment, with PCTAIRE3 showing increased staining in the nucleus and in the perinuclear area (Figure 5B). These data suggest that APP and Aβ play an equal and important role in expression of PCTAIRE-2 and PCTAIRE-3 and under pathogenic conditions the increased levels of Aβ act together with APP to exacerbate its cytotoxic effects on cells.

### Brains from AD transgenic mice and AD human show increased expression of PCTAIRE-2 and PCTAIRE-3

**Figure 6:**
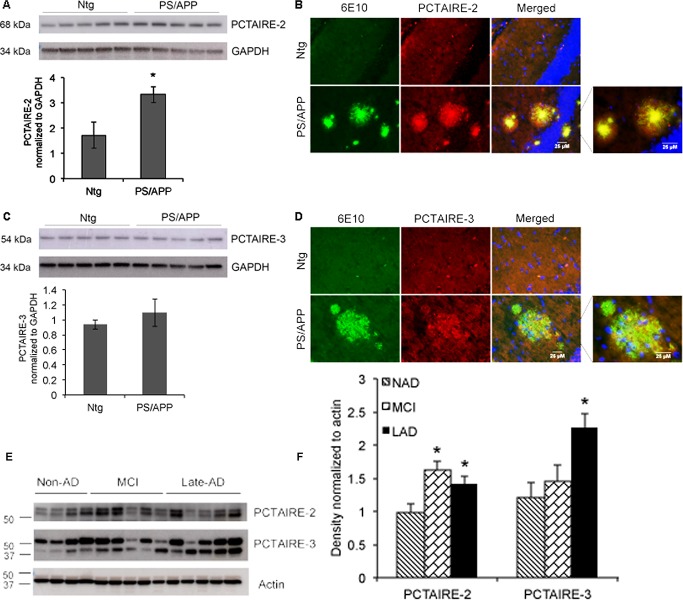
PCTAIRE-2 and PCTAIRE-3 levels in AD transgenic mice and human AD brains Brain extracts from Ntg (non-transgenic) and PS/APP mice were analyzed using **A.** PCTAIRE-2 and **C.** PCTAIRE-3 antibodies. PCTAIRE-2 showed a significant increase in the PS/APP mice. GAPDH was used to normalize the blots and ratios are shown in the bar graphs. Co-immunostaining analysis of brain sections from the mice using 6E10 (recognizing full-length APP and Aβ) and PCTAIRE antibodies reveals increased localization of **B.** PCTAIRE-2 and **D.** PCTAIRE-3 to amyloid plaques in PS/APP transgenic mice. **E.** Analysis of brain lysates from human NAD, MCI and LAD samples show increased levels of PCTAIRE-2 in MCI and LAD and PCTAIRE-3 in LAD. Actin was used for normalization and the intensity ratios are shown in the bar graph. *p value < 0.05.

Our results show that expression of both PCTAIRE-2 and 3 are significantly increased in B103-695 cells compared to APP-null B103 cells and primary neurons treated with oligomeric Aβ. These data suggest that either APP or its pathogenic Aβ metabolite is able to induce the expression of these cdks. To test if these changes occur *in vivo* we analyzed brain samples from AD transgenic mice as well as those from non-AD, MCI and late AD human subjects (the specifications of the human samples are provided in Table 1). The brains from 9 month old PS/APP double transgenic AD mice were examined by western blot and immunostaining analyses using both PCTAIRE-2 and PCTAIRE-3 antibodies. PS/APP transgenic mice demonstrate accelerated plaque pathology and increased accumulation of Aβ42 in the cerebral cortex and hippocampus at 6 months age [37]. PS/APP transgenic mice showed a significant increase in PCTAIRE-2 expression compared with non-transgenic mice, while PCTAIRE-3 expression was only slightly increased (Figure 6A and 6B). Immunostaining analysis for PCTAIRE-2 and PCTAIRE-3 also revealed increased expression in PS/APP mice compared to their non-transgenic (non-Tg) littermates (Figure 6C and 6D) and showed strong localization of PCTAIRE-2 staining to the dense core of the amyloid plaques, as detected by co-staining with 6E10 antibody (Figure 6C). Similar to the results from western blot analysis, PCTAIRE-3 staining was weaker in PS/APP mice as compared to PCTAIRE-2, and it showed punctate staining within the amyloid plaques in these brain sections (Figure 6D). Signifying the results from the cell lines, neurons and mouse models of AD, analysis of human brain samples from MCI and AD patients showed that PCTAIRE-2 and PCTAIRE-3 levels are significantly increased in AD individuals (Figure 6E and 6F). PCTAIRE-2 expression was also significantly increased in MCI brain, suggesting that this particular cdk might play a relevant role in disease advancement.

## DISCUSSION

**Figure 7:**
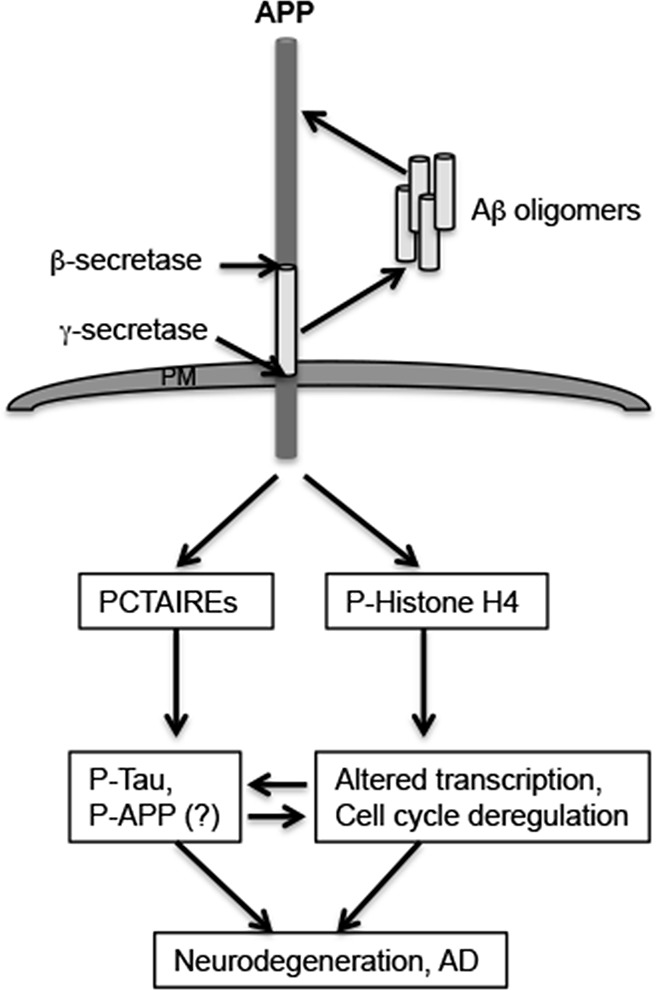
Potential APP or Aβ-mediated mechanisms in neurodegeneration The figure shows a schematic of the mechanism by which APP or Aβ may be inducing neuropathology development in AD. The results described here imply that APP or Aβ (in an APP-dependent manner) induces phosphorylation and/or expression of proteins that play a role in cell cycle (PCTAIREs) or transcriptional (P-Histone H4) dysregulation in neurons. This could potentially lead to additional phosphorylation on Tau or APP, contributing to neurofibrillary tangle formation or plaque pathology development, or promote expression of genes that play a role in AD pathology development. Interfering with these signaling cascades might prevent APP-dependent activation and promotion of neurodegeneration by these downstream targets of APP.

The results presented here show identification of novel downstream targets of APP and/or Aβ that might play a role in APP-dependent neurodegeneration in AD. The results from this study are summarized in a schematic shown in Figure 7. A comprehensive phosphoproteomic analysis of B103 and B103-695 cells resulted in the identification of both changes in phosphorylation and expression of proteins in response to APP expression. Selected, significant differentially phosphorylated proteins listed in Table 2 shows that several of these proteins are associated with cell division cycle. For example, AKAP12 has been shown to physically bind cyclin D1 in the cytoplasm and inhibit its nuclear translocation, which is necessary for its function in cell cycle progression [38, 39]. AKAP12 is phosphorylated at Ser507/515 by protein kinase C (PKC) and this modification has been shown to disrupt the cyclin binding motifs present on AKAP12 [38]. Our finding that Ser507 phosphorylation on AKAP12 is enhanced upon APP expression implies that APP mediates inactivation of AKAP12, thereby allowing nuclear translocation of cyclin D1 and G1 progression.

Phosphoproteome analysis of B103-695 cells also revealed increased phosphorylation of heat shock protein beta 1 (HspB1) at Ser15 (3.16-fold) and Ser86 (4.49-fold). The HpsB1 protein is a molecular chaperone that belongs to a family of survival proteins that modulate cell proliferation and cytoskeletal reorganization [28-30]. The phosphorylation status of HspB1 is thought to dictate both its structure and function [40, 41] and has been shown to occur in response to activation of a number of kinases [40]. An *in vitro* study using dorsal root ganglion sensory neuron model has shown that phosphorylation of HspB1 can induce cytoskeletal reorganization and neurite outgrowth [42]. In another study, stress stimuli were shown to promote phosphorylation of HspB1 at Ser15 and Ser86 and influence its subcellular localization in hippocampal rat neurons, increasing its recruitment to dendrites and synaptic sites [43]. APP is known to induce neurite outgrowth in B103 cells [19] and it is possible that this is partly mediated through enhanced phosphorylation of HspB1. APP expression was not always associated with enhanced phosphorylation on proteins. For example, B103-695 cells showed a significant reduction in the phosphorylation of myristoylated alanine-rich C-kinase substrate (MARCKS) at multiple sites. MARCKS is phosphorylated by kinases such as PKC and CaM-Kinases and has been shown to elicit cell type specific phosphorylation changes in the AD brains, where neurons showed a decrease and microglia showed an increase in phosphorylation [32]. The mechanism behind the reduced phosphorylation of MARCKS observed in B103-APP cells is unclear at this point.

Phosphorylation of Histone H4 at Ser47, which was significantly increased in B103-695 cells compared to B103 cells is novel, has not previously been reported in AD, and this was confirmed by western blot analysis. Histone H4 Ser47 phosphorylation has been shown to regulate nucleosome assembly, promoting assembly of H3.3-H4 by the histone chaperone HIRA, while inhibiting CAF-1 mediated assembly of H3.1-H4 [34]. While Histone variant H3.3 differs from H3.1 by only 5 amino acids, the functions of H3.3 are unique and cannot be substituted by H3.1 [44-46]. H3.3 is localized to gene bodies of actively transcribed genes, and this positively correlates with gene expression [47, 48]. Ser47 on Histone H4 is phosphorylated by Pak2, a member of the p21-activated serine/threonine kinase (Pak) family [34]. Additionally, phosphatases PP1α, PP1β, and Wip1 also regulate P-Ser47-Histone H4 levels [49] through activation of Pak2; depletion of PP1α and PP1β result in increased Pak2 phosphorylation at Ser141 [35, 49]. Pak2 phosphorylation at Ser141 was identified in our phosphoproteomic dataset but it was not significantly increased in B103-695 cells, suggesting that additional mechanisms are involved in the observed increase in P-Ser47 Histone H4. LAD human brain samples showed a significant increase in phosphorylation of Histone H4 at Ser47, whereas NAD showed no specific phosphorylation and MCI showed slightly enhanced phosphorylation. Furthermore, increased phosphorylation was also observed in Aβ-mediated signaling is treated primary neurons, which provides strong evidence that APP and/or Aβ are involved promotion of Histone H4-Ser47 phosphorylation. While the significance of this in AD progression has not been determined, this data suggests that APP and its metabolites may influence Histone H4-associated gene function, which may have consequences in AD.

PCTAIRE-2 and PCTAIRE-3 were also differentially phosphorylated in B103-695 cells compared to APP-null B103 cells, and this was confirmed by western blot analysis. Phosphorylation of PCTAIRE-2 at Ser146 and Ser180 has been identified in previous phosphoproteomic studies of human tissue [50-52]. Phosphorylation of PCTAIRE-3 at Ser66 has been shown previously in rats [53]. Additionally, phosphorylation of PCTAIRE-3 at Ser12, Ser66, and Ser109 has been shown in HEK293T human cells [54]. We did not confirm the changes in levels of phosphorylation at these residues due to lack of phospho-specific antibodies, only total protein expression was examined.

PCTAIRE-2 expression was significantly increased in PS/APP transgenic mice, while PCTAIRE-3 showed only slight increase. Immunostaining analysis of the Tg-AD mouse brain slices showed that both PCTAIRE-2 and 3 were localized to the dense, amyloid plaques suggesting a possible role for these kinases in Aβ-dependent pathology development. This is supported by the results from the primary neurons treated with oligomeric Aβ42, which showed an increase in expression of these two proteins, upon both immunostaining and western blot analyses. Furthermore, the finding that the changes in PCTAIRE expression and localization were more prominent in Aβ-treated APP expressing B103 cells imply that APP might act as a receptor for Aβ mediated signaling and deregulation of these less characterized Cdk family members. The findings that MCI brains show increased expression of both PCTAIRE-2 and PCTAIRE-3 and LAD brains show an increase in both PCSTAIRE-2 and PCSTIARE-3 may imply that PCSTAIRE-2 may act early in the development of the disease and persists through the progression while PCTAIRE-3 is associated with the late stage pathological changes in AD.

PCTAIRE kinases are categorized by a serine to cysteine mutation in the PSTAIRE cyclin binding consensus motif of the cell cycle associated well known CDKs [55]. PCTAIRE-2 and PCTAIRE-3 are Cdc-2-related serine/threonine kinases; however their functions remain to be discovered. Whether PCTAIREs are involved in cell cycle regulation or are regulated by the cell cycle is yet to be determined. A study by Meek and colleagues (2004) identified PCTAIRE-2 and PCTAIRE-3 as 14-3-3 binding partners, and furthermore PCTAIRE-2 interacted with 14-3-3 in a cell cycle-regulated manner. Other studies, however, indicate that PCTAIREs may function independently of the cell cycle [56, 57]. PCTAIRE-2 is expressed in terminally differentiated neurons and has been found to phosphorylate Ser and Thr residues of Histone H1 [58]. PCTAIRE-3 is expressed in the brain and testis. A study by Herskovits and Davies (2006) found increased levels of PCTAIRE-3 in the temporal cortex of AD brains compared with control brains, where it co-localized with paired helical filaments (PHFs). Furthermore, they showed that PCTAIRE-3 is indirectly involved in promoting phosphorylation of tau at residues T231 and S235, early modifications in AD pathogenesis. A separate study recently found that PCTAIRE-3 can be activated through association with Cyclin A and/or phosphorylation by Protein Kinase A (PKA) [54]. PKA increased phosphorylation of PCTAIRE-3 at Ser12, Ser66, and Ser109, though only phosphorylation of Ser12 appeared to increase kinase activity; the function of PCTAIRE-3 phosphorylation at S109 and S66 by PKA is still unknown [54]. Interestingly, PKA was also found by Herskovits and Davies (2006) in the same PHF fractions that PCTAIRE-3 was present. Bioinformatic analysis of our previous proteomic study of B103 and B103-695 cells have shown increased PKA signaling in B103-695 cells [18] and the increased phosphorylation of PCTAIRE-3 at S109 and S66 in B103-695 cells in this phosphoproteomic dataset, imply a role for PKA in the observed phosphorylations on PCTAIRE.

While further investigation is required to determine the functional role of the specific phosphorylations or expression changes in proteins described here to AD neurodegeneration and pathology development, the comprehensive phosphoproteomic dataset, together with the validation studies, provide insights into pathways that may be affected by APP expression, thereby providing a foundation for future mechanistic studies. Based on the results from A-β treated neurons, AD transgenic mice, and brain samples from AD and MCI patients, we hypothesize that the increased expression or posttranslational modifications of these identified proteins may play a relevant role in AD pathogenesis and timely inference with these modifications could prevent further progression of AD.

## MATERIALS AND METHODS

### Materials

B103 and B103-695 rat neuroblastoma cells were obtained from Dr. David Schubert (Salk Institute, La Jolla, CA). Tissue culture reagents, Hoechst 33258, and Alexa Fluor 488 and 594 dyes were purchased from Life Technologies (Carlsbad, CA). Amino acids for SILAC labeling were purchased from Cambridge Isotopes (Tewksbury, MA). Protein assay 660 reagent, ionic detergent compatibility reagent (IDCR), Halt Protease Cocktail Inhibitor, and chemiluminescence reagents were purchased from Pierce (Rockford, IL). Poly-L-Lysine (PLL) and antibodies against α-tubulin and β-actin were purchased from Sigma-Aldrich (St. Louis, MO). Anti-APP antibody (6E10) was from Covance (Princeton, NJ). Anti-Tau1 antibody and FASP 30kDa regenerated cellulose filters were purchased from Millipore (Billerica, MA). Antibodies against PCTAIRE-2 and PCTAIRE-3 were purchased from Santa Cruz (Dallas, TX), anti-GAPDH and anti-histone H4 antibodies were purchased from Cell Signaling (Danbers, MA), and anti-phosphoSer47-histone H4 antibody was purchased from Abcam (Cambridge, MA). Recombinant Aβ (1-42) peptide was purchased from American Peptide Company (Sunnyvale, CA). Sprague Dawley E16 time-pregnant rats were obtained from Harlan Laboratories (Indianapolis, IN). Anti-PHF-1 (phospho-tau Ser396/Ser404) antibody was kindly provided by Dr. Peter Davies (Albert Einstein College of Medicine, Manhasset, NY). Trypsin/Lys-C was purchased from Promega (Madison, WI) and PolyMAC phosphoenrichment kits were from Expedeon (San Diego, CA). C18 SPE desalting columns were purchased from Thermo Fisher (Waltham, MA) and strong cation exchange columns were from PolyLC Inc (Columbia, MD).

### Cell culture and SILAC labeling

B103 and B103-695 cells were grown in SILAC DMEM:F12 SILAC media supplemented with 10% dialyzed FBS, Pen-Strep-Glutamine, and either unlabeled L-arginine and L-lysine for B103 or heavy labelled ^13^C_6_-L-lysine 2HCl and ^13^C_6_-^15^N_4_-arginine HCl for B103-695 cells. Cells were grown for 5 doublings to achieve >99% incorporation of labeled amino acids before being collected.

### Sample preparation and phosphopeptide enrichment

Cells were lysed in 100mM Tris-HCl (pH 7.6), 4% SDS, 100mM DTT and Halt Protease Cocktail Inhibitor and incubated at 95°C for 5 minutes, followed by sonication at 20% amplitude. Protein was quantified using the Pierce 660 assay supplemented with ionic-detergent compatibility reagent. Experiments were performed in triplicate. A total of 1.2mg B103 and 1.2mg B103-695 lysate were combined and processed by filter-aided sample preparation (FASP) [59], followed by digestion with Trypsin/Lys-C at 1:50 (w:w; protease:protein) overnight at 37°C. Peptides were desalted using C18 SPE columns with a Supelco vacuum manifold and dried before resuspension in mobile phase A prior to fractionation. Peptides were fractionated on a Dionex U3000 HPLC system with a 200 × 4.6mm i.d. strong cation-exchange (SCX) column packed with 5μm 200Å polySULFOETHYL A-SCX material. One minute fractions were collected using a 45 minute gradient (15-200mM ammonium formate, pH 3-6.5, 25% acetonitrile) at a flow rate of 1ml/minute.

Peptide fractions were enriched for phosphopeptides using PolyMAC, a nanopolymer titanium-based enrichment, as described by the manufacturer. Following PolyMAC enrichment the samples were dried and resuspended in 0.25% formic acid for LC-MS/MS analysis.

### LC-MS/MS

Peptides were analyzed on a Q-Exactive Plus (Thermo Fisher Scientific) following with a 75μm × 50cm C18 reversed-phase(RP)-UPLC column (Dionex) using a 90 minute gradient on an EASY-nLC 1000 system (Thermo Fisher Scientific). Full MS survey scans used a resolving power of 60,000, selecting the top ten most abundant ions for MS/MS fragmentation and analysis.

### Database searching and consensus motif analysis

Raw data files were processed in MaxQuant (version 1.5.0.30, http://www.maxquant.org) and searched against the UniprotKB *Rattus norvegicus* protein sequence database. The search parameters included a constant modification of cysteine by carbamidomethylation and variable modifications of methionine oxidation and phosphorylation of serine, threonine, and tyrosine.

Statistical analysis was carried out using Perseus software (version 1.5.0.31, http://141.61.102.17/perseus_doku). Statistically significant changes in phosphopeptide abundance were determined using Significance A, an outlier test with a threshold *p*-value of 0.05. Only phosphopeptides identified in at least 2 biological replicates with a minimum ratio count of 2 were used for statistical analyses. Phosphopeptide ratios were normalized against total protein ratios from our previous SILAC-based proteomic analysis of B103 and B103-695 cells. Both non-normalized and normalized median phosphopeptide ratios were analyzed to account for potential changes in phosphorylation of proteins that were not identified in our initial proteomic analysis.

Consensus motif analysis was performed using Scaffold PTM (version 2.1.3) to determine overrepresented kinase motifs surrounding phosphorylation sites, using the method developed by Gygi and Schwartz [20], as well as potential enzyme recognition sites.

### Transgenic mouse tissue

Heterozygous PDGF-hAPP (V717F) mice (Swiss-Webster × C57BL/6) were crossed with PDGF-hPS1 (M146L) heterozygotes (Swiss-Webster × C57BL/6) to generate APP^+/−^/PS1^+/−^ genotyped mice. In this study we used these transgenic mice with age-matched non-transgenic (Ntg) mice to serve as control. Mice were anesthetized at 9 months with pentobarbital (10mg/kg body weight) and perfused with a saline solution. Brains were dissected out and half of each brain was fixed with 4% paraformaldehyde and the other half was used for protein extraction using RIPA lysis buffer. Brains were processed prior to immunohistochemical analysis as previously described [60]. Brain sections were prepared using a freezing stage microtome and then stored at 4°C in phosphate buffered saline (PBS) containing 0.02% sodium azide.

### Oligomeric Aβ42 preparation

1mg of monomeric Aβ42 was dissolved in 1ml trifluoroacetic acid and lyophilized in 100μg aliquots. Lyophilized Aβ42 was solubilized in sterile DMSO to a concentration of 5mM and then diluted to 100μM in DMEM media and left a 4°C overnight.

### B103 culture and treatment

B103 and B103-695 cells were cultured in Advanced DMEM/F12 supplemented with 10% FBS and 1% Penicillin/Streptomycin at 37°C and 5% CO_2_ as previously described [61]. Cells were plated on 8-chamber slides coated with PLL at a density of approximately 5×10^4^ cells per well. After 24 hours, cells were treated with either 5μM Aβ42 or DMSO, which served as a vehicle control.

### Primary neuron culture and treatment

Primary neurons were cultured in Neurobasal Medium supplemented with 2X B-27, 1% Penicillin/Streptomycin and 2mM glutamine. Neurons were cultured in 8-chamber glass slides and 100mm cell culture dishes coated with PLL. Briefly, E18 pregnant rats were euthanized by phentobarbital injection and feti excised and placed in isotonic solution. The meninges were then removed and cortices separated. Cortices were triturated into a single cell suspension in isotonic buffer and spun down at 1500 RPM for 5 mins at 4°C. The neuronal pellet was resuspended in 2ml Neurobasal media and filtered through a cell strainer. 8-chamber slides were plated with ∼5×10^4^ neurons per well and 100mm dishes were plated with ∼6×10^6^ neurons. Neurons were fed every third day and grown for 5 days prior to treatment. Neurons grown on 100mm dishes were treated with either DMSO vehicle or 5μM Aβ42 and harvested after 24 hrs. Neurons grown on 8-chamber were treated with wither DMSO vehicle or incremental concentrations of Aβ42 ranging from 1μM to 5μM and used for immunostaining analysis.

### Nuclear fractionation

B103 and B103-695 cells were grown in DMEM:F12 as described above, collected and pelleted by centrifugation at 500 × g for 15 minutes at 4°C. Cells were resuspended in 1ml of 10mM Tris-HCl (pH 7.4), 1mM EDTA, 200mM sucrose, and Halt Protease Inhibitor Cocktail and subjected to gentle dounce homogenization. Nuclei and cell debris were pelleted by centrifugation at 900 × g for 10 minutes at 4°C. The nuclei containing pellet was lysed in 100mM Tris-HCl (pH 7.6), 4% SDS, 100mM DTT and Halt Protease Cocktail Inhibitor as described above. Protein was quantified using the Pierce 660 assay supplemented with ionic-detergent compatibility reagent before preparing 1μg/μl samples in Laemmli Buffer for western blot analysis.

### Western blotting

Proteins were selected for validation by western blot analysis based on significance as well as function. Proteins were separated on an AnyKD SDS-PAGE gel (BioRad) and transferred to a PVDF membrane using the Trans Turboblot system (BioRad). Membranes were blocked in 5% non-fat milk in TBS for 1 hour at room temperature. Primary antibodies specific for phospho-Serine47-Histone H4 (rabbit polyclonal, 1:500), Histone H4 (mouse monoclonal, 1:1000), PCTAIRE-2 and PCTAIRE-3 (rabbit polyclonal, 1:1000), actin (mouse monoclonal, 1:7000) and GAPDH (rabbit monoclonal, 1:5000) were diluted in 5% BSA-TBS, containing 0.05% sodium azide and incubated overnight at 4°C. Membranes were washed and then incubated with appropriate corresponding secondary antibodies, donkey anti-rabbit HRP-conjugated or goat anti-mouse HRP-conjugated for 1.5 hours at room temperature. After further washes, all blots were developed with Pico Chemiluminescence reagents, with the exception of pS47-Histone H4 which was developed using Femto Chemiluminescence reagents, using an Amersham Imager 600RGB (GE Healthcare).

### Immunostaining

For immunostaining analysis of mouse brain sections, sections were mounted onto superfrost slides and rehydrated with TBS for 5 minutes. For antigen retrieval, sections were incubated in 10mM citrate buffer, pH 6.0 for 10mins at 95°C and cooled to room temperature. After washing with PBS, sections were incubated with blocking buffer (10% normal goat serum (NGS) in TBST with 0.02% sodium azide) for 2hrs at room temperature. Sections were then incubated with APP (6E10) primary antibody (mouse monoclonal 1:500) and either PCTAIRE-2 (1:50) or PCTAIRE-3 (1:50) primary antibody diluted in 1% BSA-TBST at 4°C in a humidified chamber overnight. Next, sections were washed and incubated for 2hrs at room temperature with goat anti-mouse IgG Alexa Fluor 488 (1:1000) and goat anti-rabbit IgG Alexa Fluor 594 (1:4000) diluted in blocking buffer. After washing, slides were incubated with 1μg/ml Hoechst 33342 DNA dye in PBS for 3mins. After thorough washing, the slides were coverslipped with Fluoro-Gel mounting media and analyzed with a Zeiss Fluorescence Axio Imager using AxioVision Rel 4.8 software.

For immunostaining analysis of cultured cells, they (either B103, B103-695 or primary neurons) were fixed with 4% paraformaldehyde for 10mins at room temperature and washed with PBS. Following this, cells were blocked in blocking buffer for 1hr. B103 and B103-695 cells were then incubated overnight at 4°C with α-tubulin (mouse monoclonal, 1:1000) and either PCTAIRE-2 (1:50) or PCTAIRE-3 (1:50) primary antibodies diluted in 1%BSA-TBST containing 0.02% sodium azide. Neurons were incubated with Tau 1 (mouse monoclonal, 1:500) and either PCTAIRE-2 (1:50) or PCTAIRE-3 (1:50) primary antibodies. After incubation, cells were washed and incubated in goat anti-mouse IgG Alexa Fluor 488 (1:1000) and goat anti-rabbit IgG Alexa Fluor 594 (1:4000) diluted in blocking buffer. After brief washing, cells were incubated for 3mins with 1μg/ml Hoechst 33342 DNA dye. Cells were thoroughly washed and mounted using Fluoro-Gel before being visualized and analyzed as mentioned above.

### Human brain tissue

Human brain tissue was obtained from Dr. David Cribbs at the University of California Irvine Alzheimer's Disease Research Center. Brain samples were de-identified and categorized based on post-mortem Braak stage and pre-mortem clinical MMSE score. Additional information on this brain material is detailed in Table 1. Samples were categorized based on disease stage; Non-AD (NAD), Mild Cognitive Impaired (MCI) or late-AD (LAD). Tissue was homogenized in 100mM Tris-HCl (pH 7.6) containing 4% SDS, 100mM DTT and Halt protease inhibitor cocktail, heated at 100°C for 5mins, briefly sonicated and centrifuged for 15mins at 14,000 RPM. The soluble supernatant fraction was then separated from the insoluble pellet, protein concentrations were determined using Pierce 660 reagent and equal amounts of proteins were used for sample analysis by western blot.
